# Assessment of knowledge on sexually transmitted infections and sexual risk behaviour in two rural districts of Bhutan

**DOI:** 10.1186/1471-2458-13-1142

**Published:** 2013-12-06

**Authors:** Kunzang Norbu, Sontosh Mukhia, Tshokey

**Affiliations:** 1 Medical Officer, Basic Health Unit, Gasa Dzongkhag, Bhutan; 2 Medical Officer, Basic Health Unit, Zhemgang Dzongkhag, Bhutan; 3 Clinical Microbiologist, JDW National Referral Hospital, Thimphu, Bhutan

**Keywords:** STI, Knowledge, Risky behaviour, Gasa, Zhemgang, Bhutan

## Abstract

**Background:**

The incidence of STI is high and increasing in Bhutan. Poor understanding of risky sexual behavior could be a cause. Comprehensive community surveys have not been previously done. This study was conducted to assess local knowledge on STIs and sexual risk behaviour in two rural districts of Bhutan: Gasa and Zhemgang.

**Methods:**

The study population included residents aged 15–49 years in the two districts. Health Assistants (HAs) visited all households to distribute questionnaires assessing understanding of knowledge on STIs and risk behaviour. Questionnaires were scored and analyzed.

**Results:**

The average score was 61.6%. Respondents had highest knowledge about prevention and lowest about disease and complications. There was a positive correlation between level of education and knowledge on STI (P < 0.05). Almost 37% of students scored low. Nearly one-third of the study population was practicing risky sexual behavior with 31.2% having sexual relationships with non-regular partners and 10.9% had extramarital sexual contacts. Regular use of condoms with non-regular partners was 49.1%. The most common reason for not using condom was unavailability during the sexual encounter. The study showed that despite increasing knowledge there was no reduction in risky sexual behaviour (p > 0.05).

**Conclusions:**

The study population had variable understanding of STIs and their complications. One in three persons practiced risky sexual behaviour, higher in men. Condom use was low. There was no reduction of risky sexual behaviour with increasing level of knowledge indicating that increasing level of knowledge does not necessarily reduce risky sexual behaviour.

## Background

Sexually transmitted infections (STIs) are infections with significant probability of transmission by means of sexual contact through vaginal, oral and anal sex. It is estimated that more than 340 million new cases of curable STIs occur every year throughout the world in men and women aged 15–49 years [1]. The largest proportion of these infections occur in the region of south and south-east Asia, followed by sub Saharan Africa, Latin American, and the Caribbean [1]. Since 1995, there has been increasing trend of STIs [2]. There are multiple factors that could have lead to this increasing trend. Wide spread use of oral contraceptives and other methods of contraception has lead to reduced use of barrier methods of contraception thereby exposing individuals’ risk to contact STIs. Increasing number of mobile population both within and between countries, recreational drug use, alcohol, and frequent partner changes could have lead to risky sexual behaviour. Studies have shown that lack of information and negative attitudes encourage sexual risk behaviour, thus increasing risk of acquiring STIs [3]. Available data have shown that there are higher rates of STIs between 15–44 age group [4]. These diseases may lead to serious complications such as infertility, ectopic pregnancy, cervical cancer, fetal wastage, and even death.

STIs have become a major public health issue in Bhutan in recent years [5]. Despite diagnostic, therapeutic and preventive advances, STI cases keep increasing in the country each year [6]. Questions as to why this is occurring still remain unanswered. Small surveys done in few selected areas showed that both males and females have multiple sexual partners, and extramarital sexual practices are common with promiscuity reported among both males and females [7]. Sexual exposure occured at a young age [7]. Studies have shown that despite having good knowledge about condom, its usage is not high [8]. This suggest that the community risk of contacting STIs is very high. However, no comprehensive study to asses knowledge of this risk group and their risk behaviour has been done previously.

This study was conducted to assess knowledge on STIs and community based sexual risk behaviour among the people of two high STI prevalent districts of Bhutan; Gasa and Zhemgang where the STI cases keep increasing over the past few years [6]. The study is aimed at finding out why and how the STI cases keep increasing, despite strategies taken to combat STIs by public health effort and accordingly forming an evidence based plan to effectively curb spread of disease.

## Methods

This was a community based descriptive study carried out in Gasa and Zhemgang districts of Bhutan. The study population comprised of residents in these districts aged 15–49 years including the mobile population during the study period. This target age-group was selected as studies have shown that STIs are most common between 15–49 years [1,4]. The study was done from November 2010 to February 2011.

Health Assistants (HAs) from the respective Basic Health Units (BHUs) were trained as data collectors. They visited all the households in their area for data collection. Self-administered questionnaires were distributed to the study popultaion who were asked to answer a set of questions. The data collectors helped in interpreting and answering the questions for those who couldn’t read or write in English.

### Ethical consideration

Ethical clearance was granted by Research Ethics Board of Health (REBH), Ministry of Health, Bhutan. Informed written consents were obtained before participating in this study. Those who refused to give informed consent or who refused to complete the questionnaire in the process of the study were excluded. Throughout the procedure, the confidentiality of the information provided was strictly maintained.

### Statistical analysis

Data were entered using the CSpro software 4.0 version. These data were then exported to SPSS and analysed using SPSS software 16.0 version. Descriptive statistics were used to find the frequencies and percentages. The correct answers on the knowledge were coded “1” and wrong answers “0”. All the correct answers were summed and specific and overall knowledge was calculated. Those who scored more than 90% were categorized as excellent knowledge, 70-89% as good, 50-69% as basic, and below 49% as poor knowledge. Cross-tabulation and Chi-square tests were done to find association between knowledge and socio-demographic variables, risk behaviour and socio-demographic variables, and knowledge and risk behaviour.

## Results

### Demography

Gasa and Zhemgang Districts have population of 1,832 and 10,839 respectively in 15–49 years age group [9]. A total of 2286 people participated in the study of which 76.5% were from Zhemgang and 23.5% were from Gasa. Fifty three percent of the respondents were male. The majority (78.5%) of the study population belonged to the age group 15–34 years with mean age of 27 years and SD 8.4 years. The major (62.1%) proportion of the study population were married. Thirty one percent of the respondents had no formal education, 17% went for non-formal education, 31.1% went upto secondary school, rest were graduate (5.1%) and post-graduates (1%). Farmers composed about 37% of the study population while students composed 20% (Table 1).

**Table 1:** Socio-demographic variables

**Variables**	**Number (percentage)**
District	
* Gasa*	537 (23.5)
* Zhemgang*	1749 (76.5)
Gender	
* Males*	1216 (53.2)
* Females*	1070 (46.8)
Religion	
* Buddhist*	2197 (96.1)
* Hindu*	84 (3.7)
* Catholic*	4 (0.2)
Age-group	
* 15-24 years*	919 (40.2)
* 25-34 years*	876 (38.3)
* 35-44 years*	398 (17.4)
* 45-49 years*	93 (4.1)
Marital status	
* Never married*	601 (26.3)
* Married*	1419 (62.1)
* Divorced*	113 (4.9)
* Widowed*	40 (1.7)
* Living together*	113 (4.9)
Education	
* No formal education*	692 (30.3)
* Non-formal education*	389 (17.0)
* Primary school*	347 (15.2)
* Secondary school*	712 (31.1)
* Graduate*	117 (5.1)
* Post-graduate*	22 (1.0)
* PhD*	1
Occupation	
* Farmer*	837 (36.6)
* Student*	458 (20.0)
* Business*	121 (5.3)
* Civil servant*	387 (16.9)
* Monk*	68 (3.0)
* House wife*	339 (14.8)
* Laborer*	24 (1.1)
* Others*	49 (2.1)

### Knowledge on STIs

The study assessed five aspects of knowledge on STIs in the community: transmission, symptoms, clinical disease, prevention, and complications. The average score of knowledge was 61.6%. The respondents had excellent knowledge about prevention (91%) and good knowledge about transmission (70%). However, the knowledge on the symptoms of STIs was basic (60%) and poor on clinical disease (40%) and complications (47%). Of the diseases, gonorrhoea was heard by 72% of respondents, syphilis by 58%, gential herpes by 35%, and hepatitis, chancroid and chlamydia by 24% of respondents (Table 2).

**Table 2:** Knowledge on STIs

**A. Transmission**	**Knowledge (%)**
Unprotected sex	91.3
Blood transfusion	86.0
Mother to child during birth	74.9
Hugging	69.6
Sharing towels	63.5
Breast feeding	62.9
Kissing	59.5
Unprotected anal penetration	55.2
**B. Symptoms**	
Vaginal discharge	72.9
Penile discharge	71.0
Itchiness in genitalia	70.0
Genital ulcers	61.2
Painful swelling in groin	49.9
Eye discharge in newborn	36.5
**C. Diseases**	
Gonorrhoea	72.3
Syphilis	58.2
Genital herpes	35.5
Chlamydia	24.9
Hepatitis	24.7
Chancroid	23.0
**D. Prevention**	
Use of condoms	94.1
Single partner	92.0
Timely treatment	86.7
**E. Complications**	
Caner of cervix	64.9
Abnormal baby	52.4
Still birth	46.3
Blindness in children	44.4
Subfertility	36.4
Ectopic pregnancy	35.7

The community had variable sources of information on STI. Healthcare workers were the most common source of information. Radio, television, friends, mass campaign and teachers were also important sources of information while newspapers and parents provided least information (Table 3).

**Table 3:** Sources of information

**Source**	**Percentage**
Healthcare worker	93
Radio	79
Telivision	65
Mass campaign	63
Teacher	56
Newspaper	52
Parents	46

### Knowledge vs educational status and occupation

**Figure 1:**
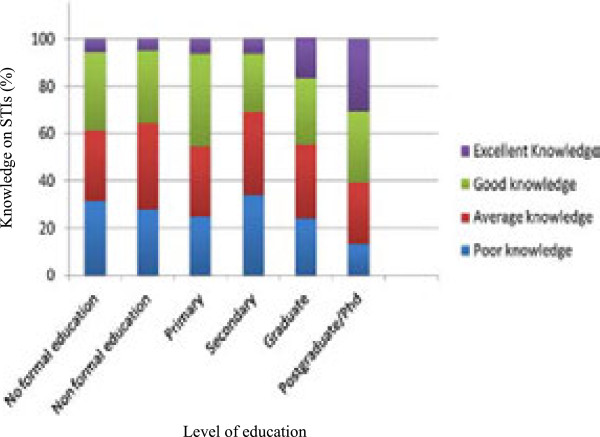
Association between level of education and knowledge.

**Figure 2:**
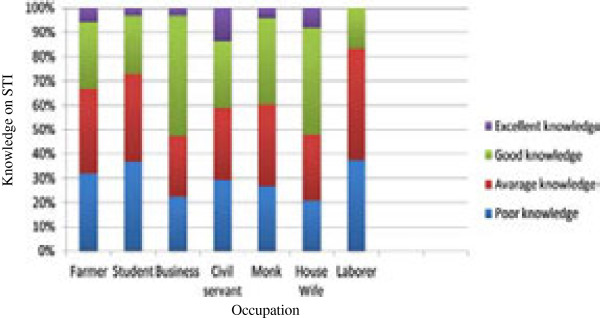
Association between occupation and knowledge on STIs.

This study revealed a statistically significant (P < 0.05) association between level of education and level of knowledge on STI. Respondents with higher educational level had more knowledge on STI (Figure 1). However, there was no significant improvement in knowledge observed when the educational level increased from primary to secondary level. This is attributed to a category of respondents who were mainly belonging to the student group and had poor knowledge on STIs. The highest level of knowledge was among the business community followed by housewives and civil servants. Laborers had the least knowledge (Figure 2).

### Sexual risk behavuour

This study had several components to assess the sexual risk behaviour: away from home, duration away from home, sexual relationship with non-regular partners in the past one year, current extramarital affairs, condom use, age of first sexual intercourse and sex before marriage (Table 4).

**Table 4:** Sexual risk behaviour

**Variables**	**Percentage**
Away from home in past one year	57.0
Duration away from home	
* Less than one week*	18.1
* Up to two weeks*	17.9
* More than three weeks*	63.0
Sexual relationship with non-regular partners	31.2
Current extramarital affairs	10.9
Condom use with regular partner	
* Always*	17.9
* Sometimes*	29.5
Condom use with non-regular partner	
* Always*	31.3
* Sometimes*	17.8
Reasons for not using condoms	
* Condom not available*	23.5
* Diminish sexual sensation*	18.7
* Cannot negotiate with partner*	15.3
* Problem of disposing*	11.2
* Not aware about importance of condom*	11.1
Age at first sexual intercourse	
* Before 15 years*	13.6
* 15-20 years*	56.6
* 20-25 years*	16.0
* 25 years or more*	3.0
* Never*	10.7
Sex before marriage	51.8

Fifty seven percent of respondents were away from home in the past one year. Amongst those, males composed 61.1%. Majority (63%) were away from home for more than three weeks. Almost one-third (31.2%) of respondents had sexual relationship with non-regular partners in the past one year and of those, 58.5% had sex with two or more different partners. At the time of this study 10.9% of the responders were having extramarital affairs. Sixty five percent of males and 37% of females had sex before marriage. Most (56.6%) of the responders had first sexual experience between 15–20 years of age and 13.6% had the experience before 15 years.

Ninety four percent of residents accepted condom use as effective method of preventing of STI. However, use of condoms during sexual contact with non-regular partners was low (49.1%). Males had higher (49.5%) condom usage compared to females (30.1%). Almost one-fourth (23.6%) of those who didnot use condom reasoned that condom was not available at the time of intercourse. 18.3% did not like using condoms since it diminished the sexual pleasure while 15.3% of the responders couldn’t negotiate with the partner in using the condom at the time of the intercourse. Regular use of condoms were 1.8 times more frequently with non-regular partners compared to regular partners (Table 4).

### Sexual risk behaviour vs knowledge and socio-demographic variables

The study showed that with increasing level of knowledge, there was no reduction in risky sexual behaviour (p > 0.05). Business community were the most mobile population in the community and they were most vulnerable to risky sexual behaviour. Seventy nine percent of the business people were away from home for at least one week in the past one year and 49.6% of them had sexual relationship with non-regular partner. Men were more mobile and 2.2 times more likely to indulge in risky sexual behaviour compared to women. It was found that risky sexual behaviour increased with duration away from home.

## Discussion

The main purpose of this study was to assess knowledge of STIs and community based sexual risk behaviours in Gasa and Zhemgang districts of Bhutan. The overall knowledge of STI in these two districts was basic (61.6%). The knowledge on prevention is excellent, however residents had very poor knowledge on clinical disease and its complications. Majority of respondents heard about gonorrhoea (73%) and syphilis (58%), while only about 24% heard about hepatitis, chancroid and chlamydia. This finding may be attributed to community perception in rural Bhutan that STIs are caused by only gonorrhoea and syphilis. A similar findings were reported from study done among university students in Kampala, Uganda [10] where gonorrhoea and syphilis were most commonly heard STI.

The community had variable sources of information on STI; healthcare workers were most common, and newspapers and parents were least common sources of information. This finding can be attributed to low literacy rate and poor access to newspapers in rural areas, and lack of culture and tradition on educating children on sexual health by parents. This finding is in contrast to study done among the college students in Kerala, India [11] which showed newspapers as the most common source of information. The finding that most (93%) of respondents had heard about STI from healthcare workers suggests that STI advocacy and education through healthcare workers in remote communities are efficient. An awareness program on HIV/AIDS and STI in these districts through establishment of Multi Sectorial Task Force (MSTF) had coverage of 63% of the residents. Therefore it is important to revisit the strategy of conducting mass campaign to target all population and provide better coverage. The finding that only 56% of respondents heard about STI from their teacher indicates that school teachers do not play important role in educating their students on STI which is further compounded by poor coverage of awareness campaign in schools.

The increasing education level showed a directly proportional level in knowledge on STI (p < 0.05). However, there was no reduction in risky sexual behaviour with increasing level of knowledge (p > 0.05). This finding is consistent with a study done in Sweden which found that higher level of knowledge was associated with increasing risky sexual behaviour [12]. National AIDS program report in 2005 showed that sexaul activity begin early with 10% having their first sexual experience by 14 years [7]. This is supported by our study as 13.6% of the respondents had their first sexual experience before the age of 15 years.

This study found that men were 2.2 times more likely to engage risky sexual behaviour than women. This supports a study conducted on sexual behaviour and networks in Thimphu by National AIDS Control Program, Bhutan and Centre for Global Public Health, University of Manitoba [13] which found that men were two times more likely than females to engage in casual or commercial sex. This can be attributed to men being more mobile population, and culture and belief that extramarital affairs by men are accepted norms in these communities.

This study showed 31.2% of respondents engaged in sex with non-regular partners in the past one year and 10.9% had extramarital sexual relationship. A study done in Nepal to assess community based risk behaviour on HIV/AIDS targeting women found that 2.2% of married and 13.7% of unmarried women indulged in risky sexual behaviour [14] which is much less than this study. More than half the study population has had sex before marriage. One third of them had sexual relationship with non-regular partners in the past one year. This finding supports the previous findings in small studies where both males and females have multiple partners, extramarital sexual practices were common, promiscuity was reported among both males and females although it was more among males [7].

Ninety four percent of respondents accepted condom use as effective way to prevent transmission of STIs. This finding is similar to a study done among elderly men in Singapore [8] which found 88.5% of respondents felt that condoms were effective way to prevent transmission of STIs. However, this study shows use of condoms during sex with non-regular partners is low. This reflects that good knowledge about condom use doesn't lead to increasing use of condoms. A knowledge, attitude and practice study conducted in Bhutan in 1989 found that only 8% amongst the 39% that were aware ever used condoms [15]. Our study shows that there are increasing percentage of people using condom (49.1%) compared to the past. There are no present national data on percentage of condom use and the finding that less than half of respondents never used condoms during sexual encounter with non-regular partners indicate that condom use is not satisfactory. Condom use was 1.8 times more common with non-regular partners compared to regular partners. Majority of those who do not use reasoned that condom was not available at the time of sexual intercourse. This finding questions how effectively the condoms are distributed to the clients despite adequate supply to all the health facilities in the country.

## Conclusion

The study population had variable understanding of STIs and their complications. One in three persons practiced risky sexual behaviour, higher in men. Condom use was low. There was no reduction of risky sexual behaviour with increasing level of knowledge indicating that increasing level of knowledge does not necessarily reduce risky sexual behaviour.

It is important to target groups possessing poor knowledge during the mass campaign, especially schools and laborers. Perhaps introduction of adolescent sexual health education in schools might play an important role as students are vulnerable to unprotected sex. Since the knowledge about diseases and their complications are poor, it is important to educate about these to make them change their risky sexual behaviour. A better and revised strategy of MSTF campaign might be needed in these districts to provide more coverage. It is also important to target high risk groups especially businessmen to reduce risky sexual behaviour. A better mode of condom distribution is also necessary to make condom available at all times. More studies in the future about the pattern of sexual behviour and sexual network in these districts might help to curb the spread of STI in such remote and vulnerable districts.

## Competing interest

The authors declare that there is no competing interest. The small amount of funding received from the HIV/STI control program, Department of public Health, Ministry of Health, Royal Government of Bhutan was used for printing of questionnaires and as salary for data collectors. There were no external agencies or private companies involved.

## Authors’ contributions

KN, SM and T jointly designed the study. KN conceived of the study and was the principal investigator. KN trained and supervised data collectors in Gasa district, analyzed data and wrote manuscript. SM trained and supervised data collectors in Zhemgang district, assisted in statistical analysis and writing manuscript. T participated in statistical analysis and writing manuscript. All authors read and approved the final manuscript.

## Pre-publication history

The pre-publication history for this paper can be accessed here:

http://www.biomedcentral.com/1471-2458/13/1142/prepub
